# The Impact of Seed Treatment with Cold Plasma on Antioxidants, Sugars, and Pigments in Needles of Norway Spruce Is Genotype-Dependent

**DOI:** 10.3390/plants14091404

**Published:** 2025-05-07

**Authors:** Ieva Čėsnienė, Vytautas Čėsna, Vida Mildažienė, Diana Miškelytė, Dorotėja Vaitiekūnaitė, Vaida Sirgedaitė-Šėžienė

**Affiliations:** 1 Laboratory of Forest Plant Biotechnology, Institute of Forestry, Lithuanian Research Centre for Agriculture and Forestry, Liepu St. 1, LT-53101 Girionys, Lithuania; ieva.cesniene@lammc.lt (I.Č.); doroteja.vaitiekunaite@lammc.lt (D.V.); vaida.seziene@lammc.lt (V.S.-Š.); 2 Department of Forest Protection and Game Management, Institute of Forestry, Lithuanian Research Centre for Agriculture and Forestry, Liepu St. 1, LT-53101 Girionys, Lithuania; 3 Faculty of Natural Sciences, Vytautas Magnus University, Universiteto 10, Akademija, LT-53361 Kaunas, Lithuania; vida.mildaziene@vdu.lt; 4 Department of Environmental Sciences, Vytautas Magnus University, Universiteto 10, Akademija, LT-53361 Kaunas, Lithuania; diana.miskelyte@vdu.lt

**Keywords:** cold plasma, *Picea abies*, chlorophyll, carotenoids, MDA, soluble sugars, phenols, flavonoids, low-temperature plasma

## Abstract

Forests face increasing threats due to climate change and anthropogenic pressures, exacerbating plant stress and disease susceptibility. Norway spruce (*Picea abies* (L.) H. Karst.), a key conifer species in European forestry, is particularly vulnerable. Developing innovative seed treatments to enhance tree resilience is crucial for sustainable forest management. Despite the growing interest in cold plasma (CP) technology for seed treatment, research on its long-term effects on trees, particularly Norway spruce, remains scarce. This study aimed to investigate the effects of pre-sowing CP treatment on Norway spruce seeds from 10 half-sib families over two vegetation seasons. Results indicate that CP treatment influenced key physiological and biochemical parameters in a genotype-specific and treatment duration-dependent manner (1 or 2 min). In some cases, CP-treated seedlings exhibited increased chlorophyll levels (e.g., increased chlorophyll *a* by up to 49% in some genotypes treated with CP for 1 min, and by up to 35% in those treated with CP for 2 min), reduced malondialdehyde (MDA) content in second-year samples (by up to 52% in some genotypes), and enhanced production of phenolics (by up to 21% in some genotypes in both treatment groups), suggesting improved stress tolerance. The 541 half-sib family is particularly noteworthy, as first-year seedlings exhibited increased levels of chlorophylls, flavonoids, and total phenols following a 2 min treatment. In contrast, second-year seedlings of the same family showed an increase in flavonoids and a reduction in MDA levels compared to the control, indicating a sustained and possibly age-modulated physiological response to CP exposure (2 min). However, responses varied across genetic backgrounds, highlighting the importance of genotype in determining treatment efficacy. These findings underscore the potential of CP technology as a tool for improving Norway spruce resilience and inform future strategies for seed enhancement in forestry.

## 1. Introduction

Climate change and anthropogenic pressures are significantly impacting forest ecosystems, exacerbating the spread of plant diseases due to shifting balances between plants, pathogens, and environmental conditions. The ongoing effects of global climate change, combined with increased globalization, heighten the risk of new disease invasions, threatening the health and productivity of forests worldwide [1,2,3]. Norway spruce (*Picea abies* (L.) H. Karst.), a prominent coniferous species in Europe, is particularly vulnerable to these pressures, including drought and pest infestations (e.g., fungal pathogens *Heterobasidion annosum* (Fr.) Bref. and *Endoconidiophora polonica* (Siemaszko) Z.W. de Beer, T.A. Duong, and M.J. Wingf.; insects like *Ips typographus* L.) making it one of the most climate-sensitive tree species [4,5,6,7,8]. Despite its susceptibility, Norway spruce remains highly valued for its wood, used in construction, furniture, and paper production, creating significant demand for high-quality timber [9,10]. In this context, advancing reforestation techniques using planting material with improved wood quality, overall resilience, and disease resistance is critical for enhancing the sustainability of spruce forest stands.

Novel technologies, such as cold plasma (CP) treatment, are being explored to improve seedling performance, seed germination, disease resistance, and overall crop performance [11,12,13,14]. Studies demonstrate that CP treatment, specifically low-temperature atmospheric dielectric barrier discharge (DBD) plasma treatment, can modulate gene expression, seed surface biochemistry, and microbial load through reactive oxygen and nitrogen species (ROS/RNS) [13,15,16], and their downstream signaling effects, potentially stimulating biochemical processes and enhancing plant growth in several species, like cotton, blueberries, and various grasses [12,17,18]. Several studies have highlighted CP’s potential in promoting seed germination, improving biomass yield, and increasing plant resistance to pathogens and stress in tomato, cotton, soybean, and red clover [11,13,19,20,21,22,23]. Specifically, seed treatment with CP has been reported to boost the production of secondary metabolites (SMs), such as phenolic compounds, which are known to contribute to plant defenses against biotic stresses in blueberries, broccoli, and basil [17,24,25]. Additionally, CP treatment has been shown to increase the synthesis of photosynthetic pigments, such as chlorophylls, in tomato and fenugreek, which are directly positively linked to plant growth and productivity [26,27]. As more species-specific responses are studied, CP is poised to become a sustainable and scalable technology in modern agriculture seed enhancement.

While much research has focused on the effects of CP treatment on seed germination and growth in herbaceous species [1], studies examining its impact on pathogen resistance and the production of bioactive compounds in trees have been scarce. Previous studies on Norway spruce have shown that CP can enhance tree seedling height and antioxidant content, especially in certain genotypes, indicating the importance of genetic variation in the response to CP treatment [28]. However, the detailed mechanisms behind these responses in trees, especially in coniferous species like Norway spruce, are severely under-researched and thus not yet fully understood.

This study aims to bridge this knowledge gap by examining the effects of pre-sowing CP treatment on distinct half-sib families of Norway spruce. We specifically focus on changes in seedling photosynthetic pigment levels, phenolic compounds, soluble sugar and lipid peroxidation (cell damage) levels over a two-year period, with the goal of better understanding the potential for CP to improve plant resilience to environmental stress. By investigating these effects in different genetic families, this study seeks to provide insights into the genotype-dependent responses to CP treatment, which could inform future strategies for improving forestry production and forest health through advanced seed treatment technologies.

## 2. Results

Biochemical analysis was performed in seedling needles from ten *P. abies* genotypes (half-sib families or individuals sharing one parent tree) growing from seeds treated with cold plasma for one or two minutes before sowing. The obtained results revealed distinct genotype- and CP treatment duration-specific responses. Almost all parameters were affected both positively and negatively depending on the treatment duration, including reduced amount of malondialdehyde (MDA), i.e., lowering the levels of the lipid peroxidation indicator. This study also indicated the differences between the two vegetative growth periods studied (first year vs. second year).

**Figure 1:**
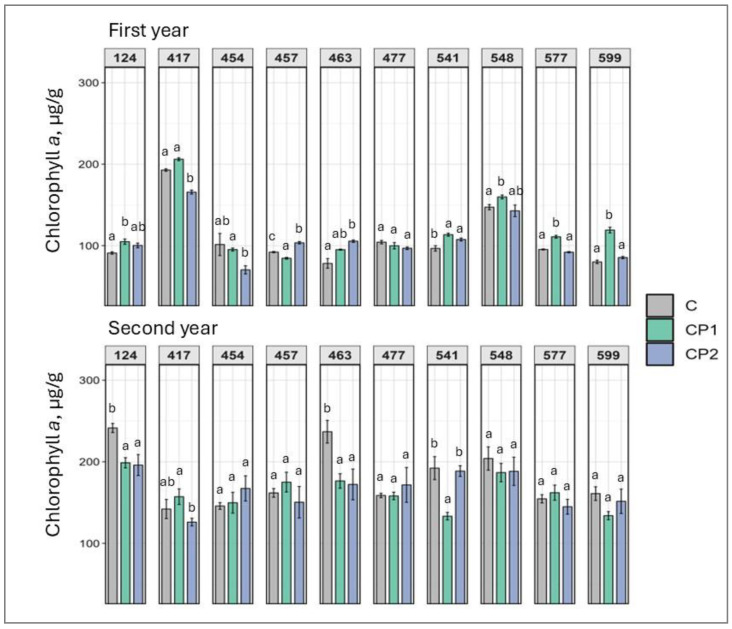
Impact of cold plasma treatment on ten Norway spruce half-sib families (genotypes indicated by three-digit numbers) chlorophyll *a* content (µg/g FW) over two growth periods (first and second year). C—control (untreated); CP1—cold plasma treatment duration of 1 min; CP2—cold plasma treatment duration of 2 min. Different letters above the columns under the same half-sib family indicate significant mean differences (*p* < 0.05), estimated using Kruskal–Wallis test followed by Dunn’s test with Bonferroni correction.

Generally, an increase in the chlorophyll levels is considered as a positive outcome, as it points to the vitality and vigor of a plant. In terms of chlorophyll *a* (Figure 1), an increase was noted in five families in the first year, treated with CP for 1 min (CP1) (from 9% to 49%) and three families where the spruce seeds were treated with CP for 2 minutes (CP2) (from 11% to 35%). This impact, however, did not translate into a similar effect for the second year. Moreover, the positive effect noted in first year with either CP1 or CP2 treatment did not occur in the same half-sib families (except family 541), indicating certain genetic aspects are in play, which determine the outcome of the CP treatment duration effect, whereby a stronger treatment duration may not necessarily lead to a greater impact in the same genotype, but different durations affect different genotypes contrastingly. Also, in both seasons, some families reacted negatively to CP treatment, with it being more pronounced in the second year. Similarly, in the second year, chlorophyll *a* levels dropped in both treatment groups in families 124 and 463.

**Figure 2:**
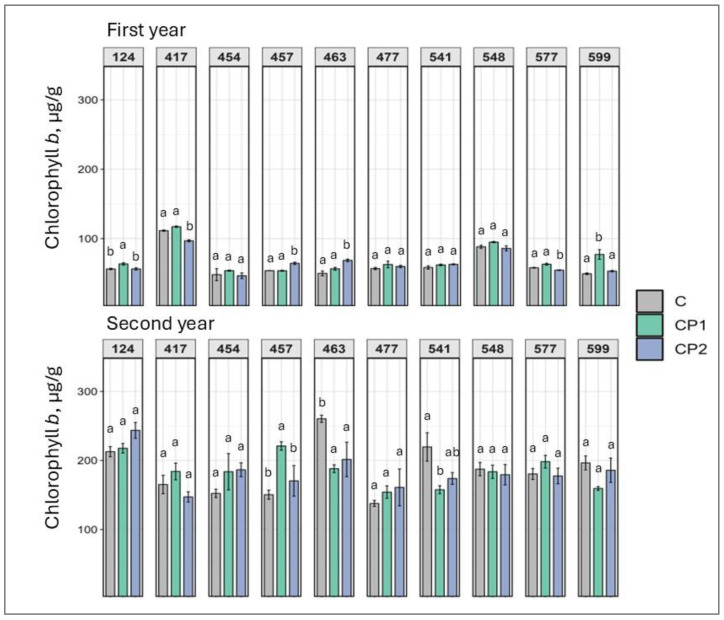
Impact of cold plasma treatment on ten Norway spruce half-sib families (genotypes indicated by three-digit numbers) chlorophyll *b* content (µg/g FW) over two growth periods (first and second year). C—control (untreated); CP1—cold plasma treatment duration of 1 min; CP2—cold plasma treatment duration of 2 min. Different letters above the columns under the same half-sib family indicate significant mean differences (*p* < 0.05), estimated using Kruskal–Wallis test followed by Dunn’s test with Bonferroni correction.

Chlorophyll *b* reacted differentially as well with regard to CP treatment (Figure 2). The levels increased in two families in the first year (from 12% to 57%) and one family (457) in the second year (by 47%) in the CP1 group, while only two families demonstrated a significant increase in chlorophyll *b* levels in the CP2 group (from 19% to 38%) (in the first year). As with the chlorophyll *a* results, these families did not overlap in terms of first year and second year. Furthermore, in some families, like 124 and 599, both chlorophyll *a* and chlorophyll *b* increased jointly (in CP1 treated group), while in family 417, they decreased jointly (in CP2 treated group) in first-year seedlings. Second year seedlings were also less likely to be significantly impacted than first year as was noted in chlorophyll *a* as well. In the second year in family 463, the chlorophyll *b* levels dropped regardless of CP dose. Also, in the 541 family, chlorophyll *b* levels dropped (by 40%) in CP1 group.

**Figure 3:**
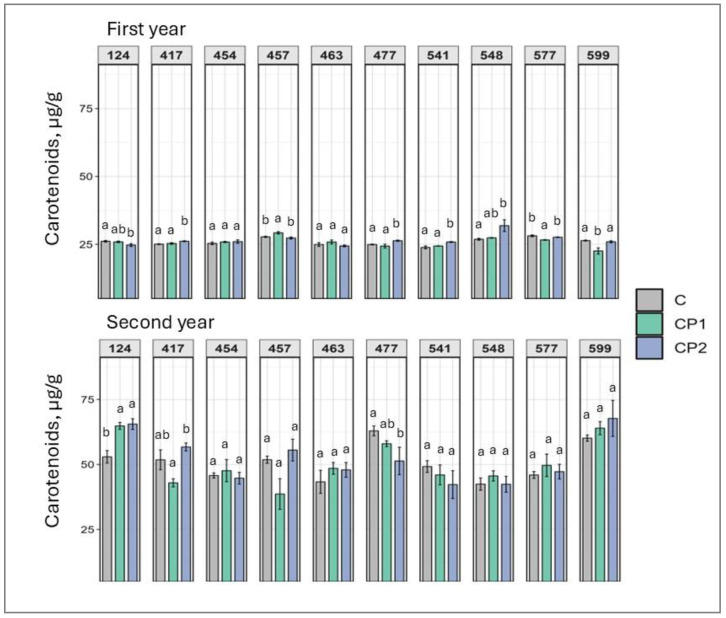
Impact of cold plasma treatment on ten Norway spruce half-sib families (genotypes indicated by three-digit numbers) carotenoid content (µg/g FW) over two growth periods (first and second year). C—control (untreated); CP1—cold plasma treatment duration of 1 min; CP2—cold plasma treatment duration of 2 min. Different letters above the columns under the same half-sib family indicate significant mean differences (*p* < 0.05), estimated using ANOVA followed by Tukey’s HSD test.

As carotenoids can serve as both pigments and antioxidants, changes in their levels should be viewed holistically, rather than individually. In the second year, only tree families were significantly affected by CP treatment in terms of carotenoid levels; in two cases, the levels increased; in one, they decreased (Figure 3). In the first year, eight families were affected, with distinct differences in response to CP duration. CP2 increased carotenoid levels in four families (from 4% to 13%), while CP1 did so in only one family (by 5%). Again, in both vegetative seasons, the seedling responses did not overlap in terms of carotenoids. Dose-dependent response to CP was noted in family 477, where carotenoid levels dropped in the CP2 group, while shorter treatment (CP1) did not affect carotenoid levels in second years seedlings.

**Figure 4:**
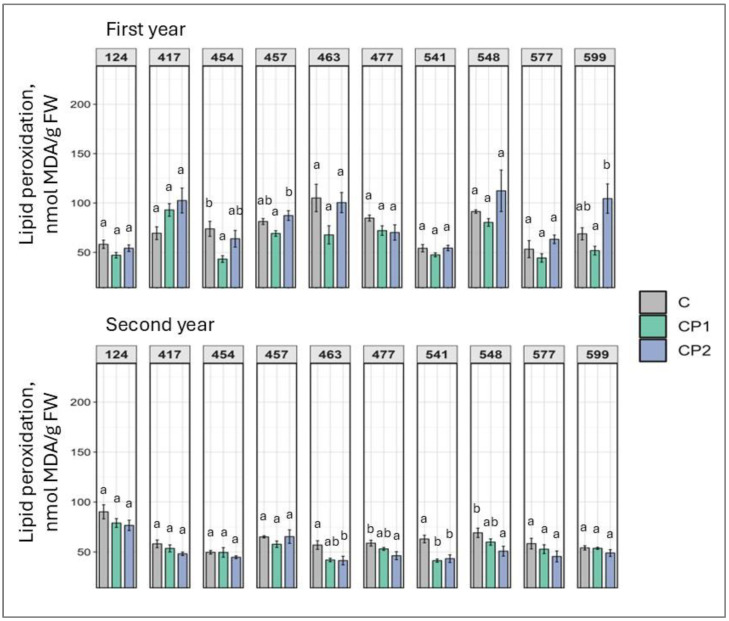
Impact of cold plasma treatment on ten Norway spruce half-sib families (genotypes indicated by three-digit numbers) lipid peroxidation indicator malondialdehyde (MDA) content (nmol/g FW) over two growth periods (first and second year). C—control (untreated); CP1—cold plasma treatment duration of 1 min; CP2—cold plasma treatment duration of 2 min. Different letters above the columns under the same half-sib family indicate significant mean differences (*p* < 0.05), estimated using Kruskal–Wallis test followed by Dunn’s test with Bonferroni correction.

MDA levels were unchanged in many cases, as seen in first year, where only family 454 under CP1 treatment experienced a decrease in MDA levels by 18% (Figure 4). Similarly, in the second year, MDA levels dropped in four families, mostly under CP2 treatment (from 27% to 52%), while in family 541, a dose-independent response was demonstrated. As elevated MDA levels indicate cell damage due to lipid peroxidation, this is a positive result overall.

**Figure 5:**
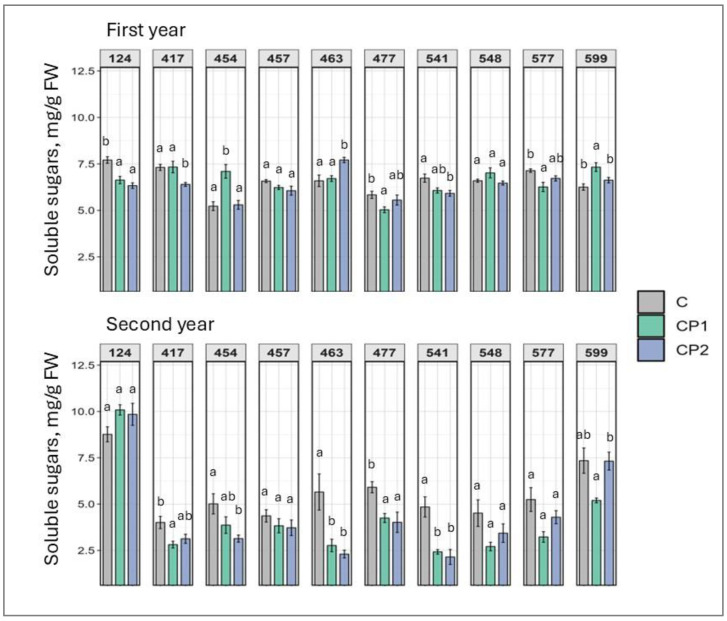
Impact of cold plasma treatment on ten Norway spruce half-sib families (genotypes indicated by three-digit numbers) soluble sugar content (mg/g FW) over two growth periods (first and second year). C—control (untreated); CP1—cold plasma treatment duration of 1 min; CP2—cold plasma treatment duration of 2 min. Different letters above the columns under the same half-sib family indicate significant mean differences (*p* < 0.05), estimated using ANOVA followed by Tukey’s HSD test.

Similarly to carotenoids, changes in soluble sugar levels can be indicative of several things, mainly an increase in the photosynthetic capacity or osmotic changes, which require sugars to act as osmolytes. The former is a positive response, while the later can serve as a stress indicator. Soluble sugar levels increased in three families (from 16% to 37%) and dropped in six families from 14% to 17% (124, 477, and 577 in CP1 group, and 124, 417, and 541 in CP2 group) in the first year (Figure 5). Family 124 again showcased a dose-independent response, while other families reacted differentially to CP treatment. In families 454 and 599 under CP1 treatment, soluble sugar levels increased, while in family 463, sugar levels increased in the CP2 group. In the second year, only decreases can be noted in five families (from 37% to 100%), in three of them regardless of CP duration. Soluble sugar levels were consistently lower during both years in the families 477 (CP1) and 541 (CP2).

**Figure 6:**
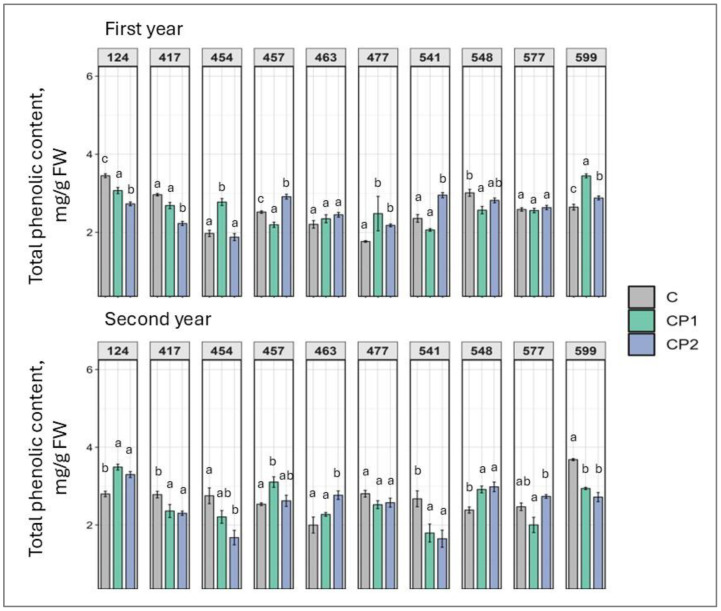
Impact of cold plasma treatment on ten Norway spruce half-sib families (genotypes indicated by three-digit numbers) total phenolic content (mg/g FW) over two growth periods (first and second year). C—control (untreated); CP1—cold plasma treatment duration of 1 min; CP2—cold plasma treatment duration of 2 min. Different letters above the columns under the same half-sib family indicate significant mean differences (*p* < 0.05), estimated using Kruskal–Wallis test followed by Dunn’s test with Bonferroni correction.

Enhanced total phenolic (TPC) and total flavonoid content (TFC) stimulate an induced systemic response, indicative of positively augmented stress response. Regarding changes in TPC, treatment duration-independent response to CP treatment was noted in the 124 (decreased), 477, and 599 (increased) families in the first year (Figure 6). In the second year, families 417, 541, and 599 demonstrated a decrease in TPC (from 17% to 69%), while families 124 and 548 demonstrated an increase (from 18% to 21%) in both CP groups. In other families, both in the first and second year, CP treatment showed a treatment duration-dependent response, e.g., CP2 was more effective in families 541 and 457, and CP1 was more effective in family 454 in the first years, and CP2 was more effective in family 463, and CP1 in family 457 in the second year.

**Figure 7:**
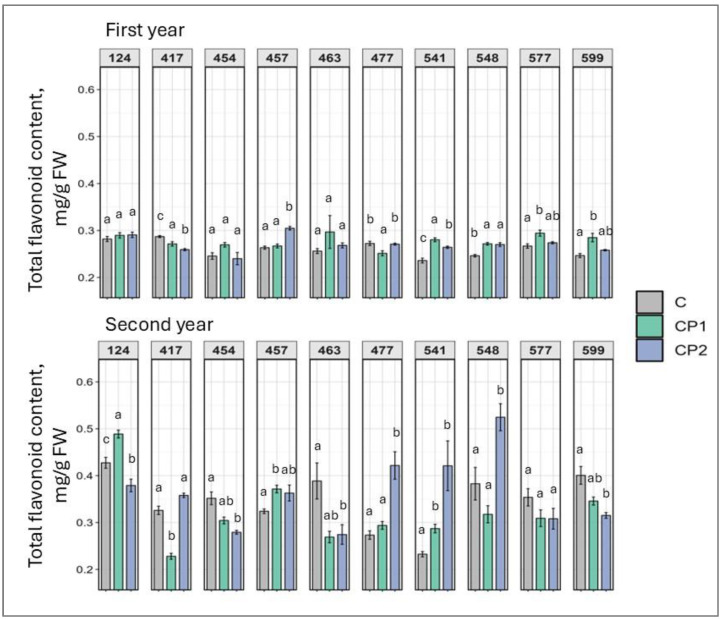
Impact of cold plasma treatment on ten Norway spruce half-sib families (genotypes indicated by three-digit numbers) total flavonoid content (mg/g FW) over two growth periods (first and second year). C—control (untreated); CP1—cold plasma treatment duration of 1 min; CP2—cold plasma treatment duration of 2 min. Different letters above the columns under the same half-sib family indicate significant mean differences (*p* < 0.05), estimated using Kruskal–Wallis test followed by Dunn’s test with Bonferroni correction.

TFC demonstrated an interesting tendency in families 541 and 548, whereby it remained elevated in first and second year (Figure 7). In family 541, the response was positive no matter the treatment duration used, while CP2 was better in family 548 (in the second year). Also, in family 417, TFC remained lower during both vegetation season in CP1 group. Otherwise, second-year seedlings exhibited both treatment duration-dependent and independent response (e.g., in the first years, five families were affected positively (from 7% to 17%) and two families negatively (from 4% to 8%), while in the second year, this number was five and five, respectively, in both CP groups.

Looking at the results collectively, several observations can be made. As mentioned, genotype- and dose-specific responses were observed. However, so were treatment duration-independent outcomes. Furthermore, responses mostly varied in first and second year. Additionally, looking at the results of both first and second years, a positive increase in TFC was exhibited by family 541 (regardless of CP treatment duration) and 548 (after CP treatment for 2 min) (Figure 7). If looking at the first year results, it can be noted that several families were impacted overall positively with marked increases in chlorophylls, TPC, TFC, soluble sugars, and carotenoids, and drops in MDA levels. Families 457, 541, and 599 particularly stand out. In family 457 (CP2), chlorophyll *a* and *b*, TPC, and TFC levels increased (Figure 1, Figure 2, Figure 6 and Figure 7). In family 541 (CP2), chlorophyll *a*, carotenoid, TPC, and TFC levels were enhanced, with no changes in MDA (Figure 1, Figure 3, Figure 4, Figure 6 and Figure 7). And in family 599 (CP1), both chlorophyll levels were increased, as were soluble sugars, TPC, and TFC (Figure 1, Figure 2, Figure 5, Figure 6 and Figure 7). As for the second year, positive outcomes were observed in family 457, whereby chlorophyll *b*, TPC, and TFC levels increased in the CP1 group (Figure 2, Figure 6 and Figure 7). Additionally, even more minor positive changes were noted in other families.

## 3. Discussion

In this study, we investigated the long-term effects of pre-sowing cold plasma (CP) treatment on Norway spruce seedlings over two vegetation seasons. We focused on assessing changes in photosynthetic pigment levels (chlorophyll *a*, *b* and carotenoids), phenolic compound content (TPC and TFC), soluble sugar concentrations, and lipid peroxidation (MDA) across 10 different half-sib families (genotypes). Our findings revealed that the responses to CP treatment were influenced by genotype, treatment duration, and seedling age.

The application of CP in seed treatment has been explored as a method to enhance seed germination [19,20,21,22], stimulate growth [20,21,22], and improve disease [23] and abiotic factor [13,19] resistance in various plant species [11]. For example, Ling et al. showed that CP treatment can improve the soybean germination rate 15%. Also, seedling growth characteristics, such as shoot length, shoot dry weight, root length, and root dry weight, exhibited notable increases of 13.77%, 21.95%, 21.42%, and 27.51%, respectively. Furthermore, the contents of soluble sugar and protein were elevated by 16.51% and 25.08%, respectively, compared to the control [20]. Jiang et al. report that plasma treatment influences the regulation of not only plant growth, but also H_2_O_2_ levels, and the activity of peroxidase, polyphenol oxidase, and phenylalanine ammonia lyase in tomato plants, enhancing their resistance to *Ralstonia solanacearum*. They conclude that as a result, CP seed treatment shows promise as a method for managing tomato bacterial wilt [23]. Moreover, it was demonstrated that CP seed treatment was effective in reducing cold-induced damage in tomato seedlings, as indicated by a higher maximum photochemical efficiency of PSII (*Fv/Fm*), along with reduced ion leakage and a lower chilling injury index [13]. All these reports generally indicate a potential beneficial application of CP on seeds as not only a way to boost growth and improve vigor but enhance resilience to biotic and abiotic factors. However, the difference in response to CP treatment duration is also noted in the abovementioned literature, as it can have negative effects as observed in the current study (e.g., reduced chlorophyll, phenolic content) and thus should be taken into account. In the current study, for example, the observed increase in the content of biologically active compounds, such as chlorophylls, did not persist in two-year-old seedlings of certain half-sib families (e.g., 124 and 463), which may be attributed to genotype-specific patterns of gene expression. Differences potentially due to variations in transcriptional activity, epigenetic modifications, or regulatory sequences could lead to distinct metabolic profiles among genotypes, thereby accounting for the age-dependent decline in compound accumulation [29]. As previously mentioned, while this study field is gaining more interest, research on trees or other woody plants is still lacking. However, some pioneering studies have reported promising results. CP treatment of tree seeds was shown to influence biochemical and growth traits that may be linked to defense responses. Specifically, in our own initial studies, in Norway spruce, exposing seeds to atmospheric DBD plasma for 1–2 min led to increased levels of phenolic compounds and photosynthetic pigments, which were strongly dependent on the genotype, with one half-sib family exhibiting accelerated seedling growth over two vegetation seasons [30]. A related study found that 1 and 2 min treatments triggered genotype-specific enhancements in antioxidant enzyme activity, including catalase, ascorbate peroxidase, peroxidase, glutathione reductase, and superoxide dismutase, as well as improved radical scavenging in seedlings aged one and two years, varying by family [28]. Conversely, research on cotton seeds applied longer plasma treatments (3–27 min), primarily affecting seed surface chemistry and water absorption [12]. In many of the studies mentioned thus far, both a genotype- and treatment duration-dependent response were observed, as were the variations in responses with plant aging. Although none of these studies directly assessed resistance to biotic or abiotic stress, the observed increases in phenolics, pigments, and antioxidant enzymes in spruce suggest that CP treatment may activate biochemical pathways related to defense. In the current study, in some half-sib families, CP induced responses independent of treatment duration (e.g., 124, 477, 599). This treatment duration-independent response may be attributed to inherent genetic traits, as these specific families exhibited similar physiological responses regardless of treatment duration—whether exposed for 1 or 2 min—indicating a genotype-specific insensitivity to CP exposure time [31].

Furthermore, the increase specifically in TPC and TFC observed in certain half-sib families in our study indicates an enhanced synthesis of secondary metabolites associated with plant defense mechanisms, following both CP1 and CP2 treatments [32]. CP treatment has been shown to modulate gene expression and increase phenolic content in various plants previously. In *Ocimum basilicum* (L.), atmospheric CP significantly upregulated genes involved in the phenylpropanoid pathway (this pathway is responsible for the production of lignin, flavonoids, phenolic acids, stilbenes, and coumarins), leading to increased essential oil content and eugenol production [24]. In blueberries, DBD CP treatment elevated levels of total phenols, flavonoids, and anthocyanins while increasing the activities of key enzymes in phenylpropanoid metabolism [17]. CP treatment of broccoli sprouts resulted in increased sulforaphane, glucosinolate, total phenol, and flavonoid content, with transcriptomic and metabolomic analyses revealing significant changes in gene expression and metabolite profiles [25]. These studies demonstrate that cold plasma can effectively modulate gene expression related to phenolic biosynthesis pathways and increase total phenolic content in plants.

Additionally, our study shows that CP treatment can enhance photosynthetic pigment levels, such as chlorophylls and carotenoids, which are directly linked to improved photosynthetic efficiency and plant productivity (more so chlorophylls than carotenoids) [33,34]. The observed increase in chlorophyll content may be attributed to CP-induced modulation of gene expression related to chlorophyll biosynthesis pathways. This could directly lead to enhanced soluble sugar levels, which were also noted in the current study [20]. CP treatment has shown promising effects on plant growth and defense mechanisms. Studies have demonstrated that atmospheric CP can enhance storage quality and inhibit chlorophyll degradation in postharvest tomatoes by regulating enzyme activity and gene expression related to chlorophyll metabolism [26]. In fenugreek, CP treatment increased chlorophyll content (up to 88%) and upregulated genes involved in diosgenin (phytosteroid) biosynthesis [27].

Although overall changes in malondialdehyde (MDA) levels following treatment were not pronounced in this study, several half-sib families exhibited a noticeable decrease in MDA content in response to CP treatment, with this effect being more pronounced in second-year seedlings. The observed reduction in MDA levels may be attributed to an enhanced antioxidant response, particularly through increased activity of key antioxidant enzymes. Our previous research demonstrated that in specific half-sib families (463, 477, and 548), the activities of catalase, ascorbate peroxidase, peroxidase, and glutathione reductase were significantly elevated following CP2 treatment [25]. Concurrently, MDA levels declined in these same families, suggesting that the upregulation of antioxidant defense mechanisms effectively mitigated lipid peroxidation induced by oxidative stress. This in part aligns with the findings of Paužaitė et al. [22], who observed that CP treatments modulated hydrogen peroxide production in germinating Norway spruce seeds, potentially contributing to improved stress responses. This was also in part corroborated by Li et al. in tomato. CP induced peroxide production, which mediated gene expression that was linked to enhanced downstream abscisic acid production [13]. The reduction in lipid peroxidation (lower MDA levels) is overall an interesting outcome of the current study, as it is commonly understood that CP induces reactive oxygen species (ROS) formation—one of CPs primary mechanisms of action. However, the researchers in the field usually link the increase in ROS to their signaling function enhancement rather than oxidative damage [11].

As noted previously and underscored by other studies, the genotype-specific responses observed in our study emphasize the importance of genetic factors in determining the efficacy of CP treatments [21,22,28]. In the current study, certain half-sib families exhibited more pronounced positive responses, suggesting that genetic selection could optimize the benefits of CP application in forestry practices.

While our study provides valuable insights into the potential of CP treatment to improve Norway spruce resilience, it is not without limitations. The exact molecular mechanisms underlying the observed changes remain to be fully elucidated. Future research should focus on detailed omics and biochemical analyses to unravel the pathways influenced by CP treatment. Additionally, long-term field studies are necessary to assess the practical applicability and sustainability of CP treatments in diverse environmental conditions.

In conclusion, our findings contribute to the growing body of evidence supporting the use of CP technology as a promising tool in forestry to enhance the accumulation of biologically active compounds in Norway spruce seedlings. By demonstrating genotype-specific responses and improvements in key physiological parameters, this study lays the groundwork for future research aimed at optimizing CP treatment protocols for large-scale forestry applications. Furthermore, future studies could include the application of direct stress factors, such as pathogen inoculation, to provide deeper insights into whether these treatments enhance tree resilience to adverse environmental conditions.

## 4. Materials and Methods

### 4.1. Sowing Material

Seeds of Norway spruce representing ten distinct genetic groups (half-sib families) were gathered from a second-generation seed orchard located in the Trakai regional division, Lithuania (54°54′38.7″ N 24°18′01.2″ E). Following collection, seeds were kept in a refrigerator (−20 °C) until treatment.

### 4.2. Treatment with Cold Plasma

Before treatment, the seeds were acclimated to room temperature for 24 h. Seeds from each Norway spruce half-sib family were subjected to cold plasma (CP) treatment, specifically low-temperature atmospheric dielectric barrier discharge (DBD) plasma. A controlled DBD plasma device, developed at Kyushu University, Japan [30], was used for treatment, ensuring a uniform exposure area of 4 × 4.38 cm^2^. The plasma treatment was applied at a discharge voltage of 7.0 kV, a current of 0.2 A, and a power density of 3.1 W/cm^2^. Two exposure durations were tested: 1 min (CP1) and 2 min (CP2). Treatments were conducted at a 5 mm distance between the seed surface and the electrode plate under atmospheric pressure, room temperature, and air humidity levels ranging from 45% to 55%. After seed treatment with CP, the seeds were left for 4 days at room temperature prior to sowing. The total number of seeds in this experiment was 4800 (10 half-sib families × 3 treatment variation × 160 seeds per treatment).

### 4.3. Growth Conditions

The seeds were sown in blocked randomized 40-cell cassettes containing a peat-based substrate (SuliFlor SF2, Sulinkiai, Radviliškis, Lithuania) with a pH range of 5.5–6.5 and were lightly covered with perlite. For the first two months, seedlings were cultivated in a greenhouse under semi-controlled conditions, where daytime temperatures ranged from 25 to 32 °C, while nighttime temperatures remained above 10 °C, with exposure to natural light. After this period, the young plants were moved outdoors to a well-lit open area. One year later, they were transplanted into containers (18 × 17 × 2 cm) filled with the same peat-based substrate (SuliFlor SF2, Sulinkiai, Radviliškis, Lithuania). Watering was provided as necessary throughout this experiment.

### 4.4. Sampling

Sampling took place twice, both times in September (2020 and 2021). The first collection was conducted before repotting to eliminate potential stress-related variations caused by the transplanting process. Samples of the seedling needles were collected by taking 3–5 needles from each of the 40 seedlings in each group. Needles were then promptly transported to the laboratory on ice. A minimum of 20 seedlings per genetic group were sampled. Three biological replicates per group were obtained (each taken from the bulk sample, thus from several individuals).

### 4.5. Biochemical Analyses

Seedling chlorophyll *a* and *b*; carotenoid; secondary metabolite (SM); total phenol (TPC) and total flavonoid (TFC); lipid peroxidation, i.e., malondialdehyde (MDA); and soluble sugar levels were measured spectrophotometrically using a SpectroStar Nano microplate reader (BMG Labtech, Offenburg, Germany) with 96-well microplates.

#### 4.5.1. Extract Preparation

For each experimental group, 0.1 g samples were ground using a tissue homogenizer (Precellys 24, Bertin Technologies, Aix-en-Provence, France) at 1956× *g* for 30 s, with the addition of two metal beads to facilitate the process. Subsequently, 1.5 mL of 80% ethanol (v/v in water, MV GROUP Production, Kaunas, Lithuania) was introduced, and homogenization was performed again under the same conditions. The homogenized mixture was then subjected to centrifugation at 16,090× *g* for 30 min at 4 °C using a Hettich Universal 32R centrifuge (Andreas Hettich GmbH & Co. KG, Tuttlingen, Germany). The collected supernatant was used further.

#### 4.5.2. TPC Analysis

To determine TPC, the extract was combined with Folin–Ciocalteu reagent (VWR International GmbH, Vienna, Austria) at a 1:9 (*v*/*v*) ratio in water and incubated for 5 min. Following this, 10% sodium carbonate was added, and the mixture was left in the dark for 1 h. Absorbance was recorded at 725 nm. The formulas and calibration methods used in this analysis have been previously described [35].

#### 4.5.3. TFC Analysis

For TFC, the extract was mixed with a reaction buffer containing absolute ethanol (Merck, Darmstadt, Germany), aluminum chloride (Alfa Aesar, Karlsruhe, Germany), potassium acetate (Sigma Aldrich, St. Louis, MO, USA), and distilled water. The mixture was incubated in the dark for 30 min, after which absorbance was measured at 415 nm. Details on formulas and calibration procedures have been described in an earlier study [35].

#### 4.5.4. Chlorophyll *a*, Chlorophyll *b*, and Carotenoid Content Analysis

Extract absorbance was measured at 470, 648, and 664 nm. The formulas and calibration details used in these calculations were previously reported [35].

#### 4.5.5. Lipid Peroxidation Analysis

Malondialdehyde (MDA) content was assessed using a modified method. The supernatant was mixed with a reaction solution containing trichloroacetic acid (Molar Chemicals Kft, Halásztelek, Hungary) and thiobarbituric acid (Alfa Aesar). The mixture was incubated at 95 °C for 30 min, then cooled on ice. Absorbance was recorded at 440, 532, and 600 nm. The formulas and methodological details were documented in a previous study [36].

#### 4.5.6. Soluble Sugar Analysis

Soluble sugars were quantified by reacting the sample with anthrone reagent (Carl Roth, Karlsruhe, Germany), prepared by dissolving anthrone in concentrated sulfuric acid (Chempur, Piekary Śląskie, Poland) according to Čėsnienė et al. [36]. The reaction mixture was incubated at 90 °C for 1 h, after which absorbance was measured at 620 nm.

### 4.6. Statistical Analysis

All statistical data were analyzed using R (Version 4.2.1) with RStudio (Version 1.1.456) programming language. Data were analyzed separately for each analyzed parameter (TPC, TFC, Chl a, Chl b, Caro, TSS, and MDA) at two different time points (first year and second year). A stepwise approach was applied to assess the effects of different seed treatment timings with cold plasma (1 and 2 min) within each plant half-sib family.

Initially, the normality of the data was assessed using the Shapiro–Wilk test [37]. If the assumption of normality was met (*p* > 0.05) (for carotenoids and total soluble sugars), a one-way analysis of variance (ANOVA) was conducted, followed by Tukey’s Honest Significant Difference (HSD) test for pairwise comparisons [38]. For datasets violating the normality assumption (for chlorophyll *a*, chlorophyll *b*, lipid peroxidation, total phenolic content, and total flavonoid content), a non-parametric Kruskal–Wallis test was performed [39], followed by Dunn’s test [40] with Bonferroni correction for multiple comparisons [41]. Significance letters indicating group differences were assigned using the multcompView package [42], based on pairwise *p*-values. Data are summarized as mean ± standard error (SE).

Graphical representations of the data were generated using ggplot2 [43] using R (Version 4.2.1) with RStudio (Version 1.1.456) programming language. Bar plots were created to display mean values of each treatment, with error bars representing SE. Significant group differences were annotated using lowercase letters positioned above the bars.

## Data Availability

Data will be made available upon request.

## Abbreviations

The following abbreviations are used in this manuscript:

CP	cold plasma
CP1	CP treatment for 1 min
CP2	CP treatment for 2 min
DBD	dielectric barrier discharge
MDA	malondialdehyde
SM	secondary metabolites
TFC	total flavonoid content
TPC	total phenolic content
